# A university-clustered tuberculosis outbreak during the COVID-19 pandemic in eastern China

**DOI:** 10.3389/fpubh.2022.978159

**Published:** 2022-08-23

**Authors:** Jizhou Wu, Limei Zhu, Jiaxi Yu, Qiao Liu, Xiaoyan Ding, Peng Lu, Yunliang Wu, Jiansheng Sun, Leonardo Martinez, Wei Lu, Jianming Wang

**Affiliations:** ^1^Department of Epidemiology, Center for Global Health, School of Public Health, Nanjing Medical University, Nanjing, China; ^2^Department of Chronic Communicable Disease, Center for Disease Control and Prevention of Jiangsu Province, Nanjing, China; ^3^Center for Disease Control and Prevention of Xuzhou City, Xuzhou, China; ^4^Department of Epidemiology, School of Public Health, Boston University, Boston, MA, United States; ^5^Department of Epidemiology, Gusu School, Nanjing Medical University, Nanjing, China

**Keywords:** tuberculosis, screening, whole-genome sequencing, preventive therapy, university

## Abstract

During the COVID-19 pandemic in 2020, a tuberculosis outbreak occurred in a university in eastern China, with 4,488 students and 421 staff on the campus. A 19-year-old student was diagnosed in August 2019. Later, the first round of screening was initiated among close contacts, but no active cases were found. Till September 2020, four rounds of screening were performed. Four rounds of screening were conducted on September 9, November 8, November 22-25 in 2019 and September 2020, with 0, 5, 0 and 43 cases identified, respectively. A total of 66 active tuberculosis were found in the same university, including 4 sputum culture-positive and 7 sputum smear-positive. The total attack rate of active tuberculosis was 1.34% (66/4909). The whole-genome sequencing showed that the isolates belonged to the same L2 sub-specie and were sensitive to all tested antituberculosis drugs. Delay detection, diagnosis and report of cases were the major cause of this university tuberculosis epidemic. More attention should be paid to the asymptomatic students in the index class. After the occurrence of tuberculosis cases in schools, multiple rounds of screening should be carried out, and preventive therapy should be applied in a timely manner.

## Introduction

Tuberculosis remains a global health problem with high morbidity and mortality (1). Although the elderly are at-risk groups for tuberculosis, teenagers and students can not be ignored (2). In recent years, the tuberculosis outbreak in schools has attracted wide attention (3–5). The number of student cases has been increasing, although the size of the national tuberculosis epidemic is shrinking (2, 6). The high attack rate was probably due to the exposure to the source of infection in a closed environment.

The ability to discern and track individual *Mycobacterium tuberculosis* (*M.tb*) strains is of critical importance to identify the source of infection and routes of transmission. With the development of biological information and the increasing maturity of sequencing technology, the whole-genome sequencing (WGS) has provided powerful tools to track the transmission and identify the drug resistance of *M.tb* (7), showing its essential role in the tuberculosis outbreak investigation (8).

During the COVID-19 pandemic in 2020, a tuberculosis outbreak occurred in a university in eastern China. Since the initial case was found in 2019, due to various reasons, the epidemic lasted for a long time until the end of 2020. In order to comprehensively analyze the epidemic process of tuberculosis on campus, we used field investigation, infection screening, and laboratory examination, especially the application of WGS technology.

## Materials and methods

### Study subjects

The outbreak occurred in a university in Jiangsu province, China. This university is located in eastern China, with 4,488 students and 421 staffs. Tuberculosis was diagnosed according to the national guideline (9), mainly based on the epidemiological history, clinical manifestation, chest X-ray examination, and laboratory tests. The tuberculin skin test (TST) was used to screen for the person infected with *M.tb*. The skin test reaction was read between 48 and 72 h after administration by a trained health care worker. The reaction was measured in millimeters of the induration and divided into ≤5 mm, 5–10 mm, 10–15 mm, and ≥15 mm (10).

### Close contacts screening

Close contacts referred to individuals who had direct contact with active tuberculosis patients. In general, all students and teachers should be included to the screening for at least once. The first round of screening covered all teachers and students in the index case class. The second round of screening included teachers and students staying in the same teaching building and living in the dormitories on the same floor, including those negative in the first round. The target population of the third screening were people who were not screened in the previous two rounds. All students and teachers in this university were screened in the fourth round.

### Laboratory test

The Ziehl-Neelsen method was used for acid-fast staining and Lowenstein-Jensen solid medium was used for culture. GeneXpert MTB/RIF (Xpert, Cepheid, USA) was performed on collected sputum samples. The sputum sample was mixed with the sample processing solution in a ratio of 1:2. Then 2 ml of the mixture was drawn into the GeneXpert MTB/RIF reaction box, and finally, the reaction box was put into the detection system (11). Four types of first-line and two types of second-line antituberculosis drugs were used to perform phenotypic drug susceptibility tests (DSTs) (12). The critical concentrations was 40.0 mg/L for rifampin (RIF), 0.2 mg/L for isoniazid (INH), 2.0 mg/L for ethambutol (EMB), 4.0 mg/L for streptomycin (SM), 40.0 mg/L for capreomycin (CM), and 2.0 mg/L for levofloxacin (LFX).

### Whole-genome sequencing analysis

Genomic DNA was extracted from cryopreserved strains and purified by Cetyltrimethylammonium Bromide (CTAB) method (13). Sequencing was performed on the Illumina Miseq (Illumina, San Diego, California) (14). The BBmap software was used to compare the sequencing reads with the reference of H37Rv (GenBank NC000962.3) (15). The minimum number of reads covering a site to be considered (default = 10), the minimum VCF variant call “quality” (default = 100). We used two online tools, “SAM-TB” (https://samtb.uni-medica.com/index) and “TB-Profiler” (https://tbdr.lshtm.ac.uk/), to obtain drug resistance and strain types from the raw sequence. Isolates with a difference of <10 pairs of single nucleotide polymorphisms (SNPs) were considered homologous (16).

### Prophylactic therapy

Students and teachers with strongly positive TST received prophylactic therapy. All active tuberculosis patients were treated with a standardized first-line regimen (2 months of Isoniazid, Rifampicin, Ethambutol, and Pyrazinamide, plus 4 months of Isoniazid and Rifampicin).

### Statistical analysis

All statistical analysis and graphing were performed through software R4.1.0 (https://www.r-project.org/). Categorical variables were expressed as percentages and analyzed using the chi-square test. -Three pies were used to compare the difference of developing active tuberculosis between index class and non-index class. A *P* value of 0.05 or less was considered statistically significant.

## Results

### Index case and the first round of screening

In August 2019, a 19-year-old student presented symptoms of paroxysmal cough, fever, sputum, and night sweats. She was diagnosed with tuberculosis pleurisy through a series of inspections and then reported to the local Center for Disease Control and Prevention (CDC). On September 9, the first round of tuberculosis screening was initiated among close contacts, including 73 students and 18 teachers. After TST and chest X-ray examinations, one person had an induration of 15 or more millimeters and no one had abnormal chest X-ray examinations.

### Subsequent cases and close contacts screening

On November 8, the second student was diagnosed with pulmonary tuberculosis after seeking health care. Then local CDC performed the second screening from November 22 to November 25, including 1,109 students and 187 teachers staying in the same teaching building and living in the dormitories on the same floor. Among them, 45 had a TST induration of 15 or more millimeters, 6 had abnormal chest X-ray examinations, and 5 were diagnosed with active tuberculosis. Of 5 tuberculosis patients detected in the second round, 2 patients were asymptomatic in the first round and had negative TST and abnormal chest X-ray examinations.

On December 15, one student, who was strongly positive in the second round of screening, took the initiative to see a doctor and was diagnosed with pulmonary tuberculosis. Then, the local CDC conducted the third round of screening using TST and chest X-ray examination from December 16 to December 18. Among 3,634 close contacts, 564 had a TST induration ≥10 mm, and 155 had a TST induration ≥15 mm (Table 1). No one showed abnormal chest images.

**Table 1:** Four rounds of close contacts screening.

	**Total**	**TST results**				**Abnormal chest X-ray (covered in previous round)**	**Suspected patients (covered in the previous round)**	**Confirmed patients (covered in the previous round)**
		**<5 mm**	**5–10 mm**	**10–15 mm**	**≥15 mm**			
**First round**
students	73	64	1	7	1	0 (0)	0 (0)	0 (0)
teachers/staff	18	13	4	1	0	0 (0)	0 (0)	0 (0)
**Second round**
students	1,109	748	174	158	29	6 (2)	6 (2)	5 (2)
teachers/staff	187	128	19	24	16	0 (0)	0 (0)	0 (0)
**Third round**
students	3,400	2,473	380	400	147	0 (0)	0 (0)	0 (0)
teachers/staff	234	196	21	9	8	0 (0)	0 (0)	0 (0)
**Fourth round**
students	4,488	2,641	948	708	191	45 (30)	45 (30)	43 (28)
teachers/staff	421	245	72	62	42	0 (0)	0 (0)	0 (0)

From August 2019 to December 2019, the diagnosed cases had been reported to the infectious disease surveillance and the two rounds screening were given within 24 h upon reporting.

In early 2020, due to the emergence of COVID-19, all students left campus and studied online at home. During January 2020 and August 2020, 15 students scattered in various districts were diagnosed with tuberculosis in local hospitals. After students returned to school in September 2020, the fourth round of screening was performed for 4,909 subjects, of which 233 had a TST induration ≥15 mm and 45 had abnormal chest X-ray images. After a series of laboratory tests, a total of 43 students were diagnosed with tuberculosis.

**Figure 1:**
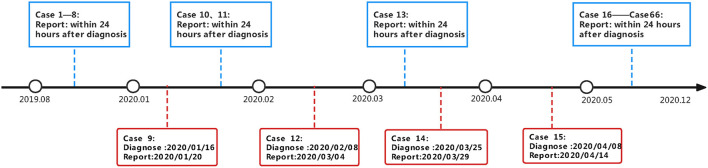
Timeline of close contacts screening and case finding in a school tuberculosis outbreak investigation.

Figure 1 showed the timeliness and delay in reporting of cases after diagnosis. From January 2020 to May 2020, 4 patients were not reported to the infectious disease surveillance system within 24 h. Meanwhile, during the COVID-19 pandemic, the public health where the students were domiciled didn't liaise with the CDC where the university was located. Due to the regulation and capacity constrains, timely and effective screening including TST and chest X-ray had not been carried out, which resulted in the delay diagnosis of asymptomatic stunents.

In total, 66 active tuberculosis patients were diagnosed in this university, of which 4 were sputum culture-positive, 7 were sputum smear-positive, and 18 were GeneXpert positive (Table 2). Phenotypic drug susceptibility tests showed that 4 confirmed isolates were susceptible to all anti-tuberculosis drugs. Three classes had highest numbers of patients and majority cases happened in grade 1.

**Table 2:** Demographic and clinical characteristics of the patients in a university, Jiangsu, China.

**Characteristics**	**Patients**	**%**
**Age (years)**
18-	8	12.1
19-	34	51.5
20-	24	36.4
**Gender**
Male	34	51.5
Female	32	48.5
**Bacteriological diagnosis**
Positive	18	27.3
Negative	48	72.7
**Sputum culture**
Positive	4	6.1
Negative	62	93.9
**Sputum smear**
Positive	7	10.6
Negative	59	89.4
**GeneXpert**
Positive	18	27.3
Negative	48	72.7
**With diabetes**
Yes	0	0.0
No	100	100.0
**Discovery method**
clinical consultation	18	27.3
contact screening	48	72.7
**TB-related symptoms**
No cough	30	45.5
Cough	36	54.5

### Attack rates of active tuberculosis

**Figure 2:**
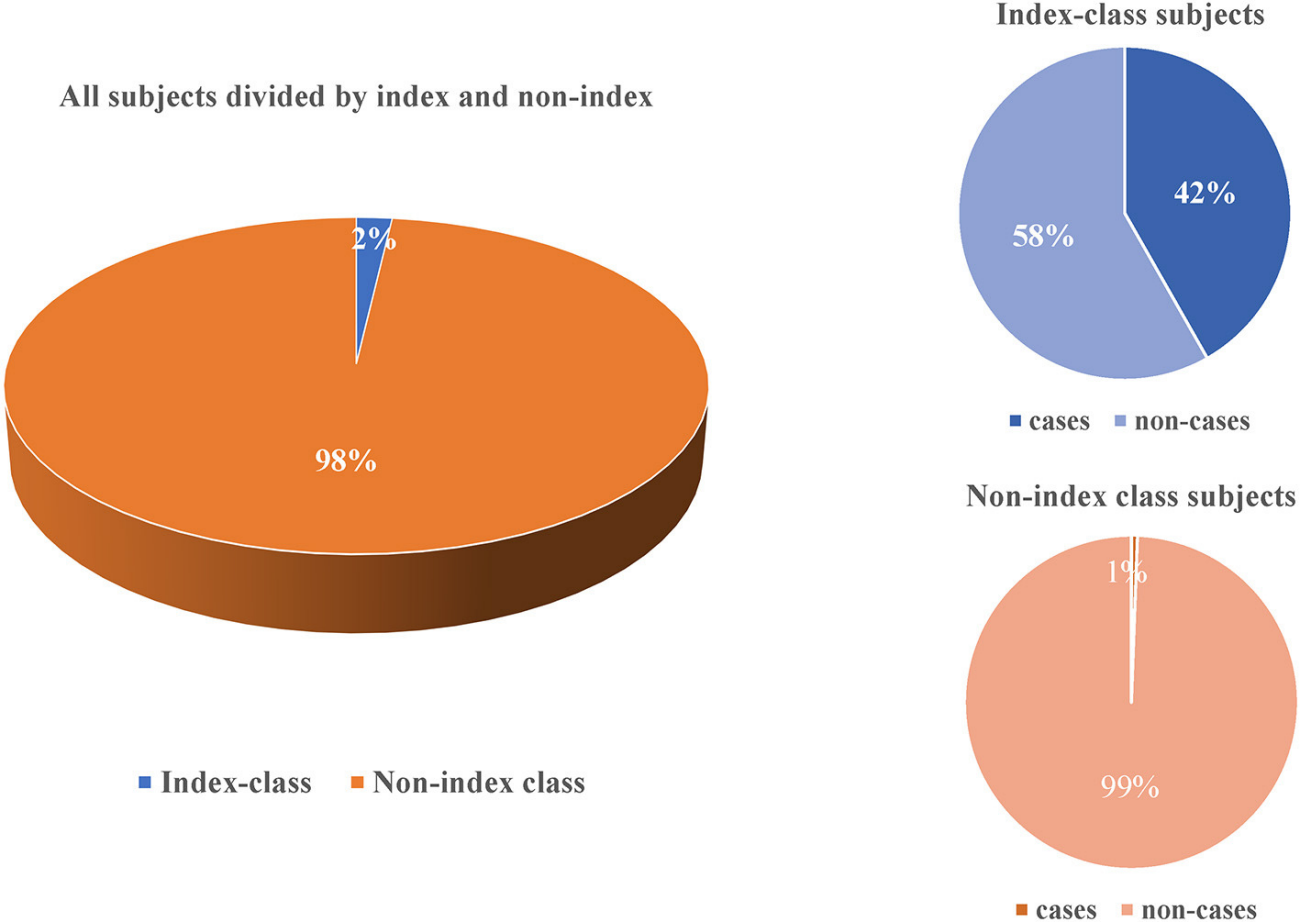
Comparison for active tuberculosis between teachers and students in the index class and non-index class in this outbreak.

The total attack rate of active tuberculosis was 1.34% (66/4909), of which 2.23% (34/1525) in male students and 0.95% (32/3384) in female students. In the index case class, the attack rate among teachers and students was 0 (0/18) and 52.05 (38/73) respectively (Table 3). Compared to the non-index class, there was a significantly higher prevalence of active tuberculosis in index class (41.76 vs. 0.58%, *P* < 0.05) (Figure 2).

**Table 3:** Attack rates of active tuberculosis in this school tuberculosis outbreak, Jiangsu, China.

**Group**	**No.at risk**	**Active TB**	
		**N**	**Attack rate (95%CI)**
Total	4,909	66	1.34 (1.04, 1.70)
Teachers	421	0	0
Students	4,488	66	1.47 (1.14, 1.87)
**Gender**
Male	1,525	34	2.23 (1.55, 3.10)
Femal	3,384	32	0.95 (0.65, 1.33)
**Classes**
Index class	91	38	41.76 (31.50, 52.57)
Non-index class	4,818	28	0.58 (0.39, 0.84)
**Teachers**
Index class	18	0	0
Non-index class	403	0	0
**Students**
Index class	73	38	52.05 (40.04, 63.90)
Non-index class	4,415	28	0.63 (0.42, 0.92)

### WGS analysis and phylogenic tree

We successfully extracted DNA from four sputum culture-positive samples. WGS showed that the distance of SNPs between four isolates was <12. These four strains belonged to the L2, and no drug resistance-related genomic sites were found.

### Treatment outcome

175 Students and 42 Teachers With Strongly Positive TST Were Given the Prophylactic Therapy. No Teachers and Students who Accepted Preventive Treatment Have Developed Active Tuberculosis.

## Discussion

The incidence of tuberculosis among general populations is continuously declining in China, but the number of student cases is rising (6). School with high population densities can be a greenhouse of pathogens, and school outbreaks of tuberculosis have been reported frequently (3, 5, 17). This study described a school-clustered tuberculosis outbreak during the COVID-19 epidemic and highlighted the importance of interventional measures to prevent a larger scale transmission.

There are several features that are worth consideration in this outbreak. First, delayed diagnosis is common in the school tuberculosis outbreaks (18). Before the detection of the first case, pathogen transmission may have been going on among students and teachers for quite a long time. Considering the window stage after infection, several screening and follow-up rounds are critical to identifying infected persons and active cases. Second, few cases were diagnosed because of active seeking health care due to the lack of apparent symptoms. As shown in Table 2, out of 66 active patients, 72.7% were detected through contact screening, and more than 45% had no tuberculosis-related symptoms. Third, during the pandemic, COVID-19 was the main concern in every clinic and may result in delaying the diagnosis and treatment of tuberculosis (19). Only 5 student patients were screened in the previous three rounds through TST and chest X-ray. Some of the asymptomatic students later developed active TB during the COVID-19. During this phase, some hospitals failed to report to the infectious disease surveillance system in a timely manner and the public health department took no actions due to the policy constrains, which caused further spread of this tuberculosis outbreak. One of our previous studies in Jiangsu Province showed that tuberculosis notifications dropped 52% during the COVID-19 pandemic in 2020 compared to 2015–2019, and the treatment completion and screening for drug resistance decreased continuously in 2020 (20). Regional lockdown, transportation restrictions, and school closures have all contributed to the delays in case detection and close contacts screening. Fourth, for fear of being stigmatized or affecting their studies, some students and their parents consciously do not report disease status and choose to go to school, resulting in continuous transmission among students.

WGS has been promoted in the investigation of tuberculosis outbreaks to determine the genotypes of *M.tb* and drug resistance. A pilot study of the European surveillance system proved that it was feasible to monitor the occurrence of multidrug-resistant tuberculosis outbreaks and could better clarify the mode of cross-border transmission (21). Compared with traditional epidemiological methods, WGS is time-consuming and can quickly determine the source of infection and route of transmission (22). However, in many investigations of tuberculosis outbreaks, it is challenging to obtain *M.tb* strains for sequencing.

For high-burden countries, it is crucial to explore safe and effective preventive treatment tools and to establish an optimized LTBI management system (23). Currently, the state encourages tuberculosis preventive therapy for students with latent TB infection. The effectiveness of preventive treatment in high burden countries has not been determined, especially for China with high MDR prevalence. To date, few studies have evaluated the tuberculosis preventive therapy (TPT) effectiveness and the probability of treatment completion in adolescents in the tuberculosis outbreak (24, 25). The directly observed therapy (DOT) and full course management (FCM) were widely used in the preventive treatment of LTBI. A study in Dalian, China, showed that DOT is effective and plays an irreplaceable role in improving preventive treatment adherence and outcomes (26). In our study, no students who accepted preventive treatment have developed active tuberculosis, and follow-up is required to evaluate the effectiveness of tuberculosis preventive treatment. It is crucial to explore safe and effective preventive treatment tools and to establish an optimized LTBI management system (23).

There were several limitations in this study. First, the proportion of bacteriological evidence in tuberculosis cases is very small, and most of them are clinically diagnosed. Previous studies have shown a low rate of pathogenic positivity in the student population and the bacterial load in children's specimens is low and difficult to collect (27, 28). To improve the sensitivity of bacteriological testing, some researchers suggest the use of multiple sampling methods for microbiological diagnosis, including serial collection of biological samples such as gastric aspirates, nasopharyngeal aspirates, sputum, bronchoalveolar lavage, and lymph node aspirates and urine (29). However, in this study, we collected sputum from patients on a continuous basis and performed pictures and cultures, which explained the low bacteriological examination. This is a common problem in most tuberculosis outbreak investigations, which has limited our understanding of the whole transmission network (30). Second, we did not follow those students who received preventive therapy for a long enough time. But we checked the national tuberculosis surveillance system in January 2022, and none of them was reported as active tuberculosis.

In conclusion, delay detection, diagnosis and report of cases were the major cause of this university tuberculosis outbreak. More attention should be paid to the asymptomatic students in the index class.After the occurrence of tuberculosis cases in schools, multiple rounds of screening should be carried out, and preventive therapy should be applied in a timely manner. WGS is the approach to support the transmission theory. This study could give a clear picture of transmission, screening-intervention, and the disruption of case finding routine due to COVID-19 pandemic and its negative impacts to TB control in university settings.

## Data availability statement

The sequencing data presented in the study are deposited in the CNCB (China National Center for Bioinformation) repository, accession number PRJCA010507.

## Ethics statement

The studies involving human participants were reviewed and approved by Nanjing Medical University. The patients/participants provided their written informed consent to participate in this study. Written informed consent was obtained from the individual(s) for the publication of any potentially identifiable images or data included in this article.

## Author contributions

JWu, LM, and JY conceived the study, analyzed the data, and drafted the manuscript. QL, JS, and YW participated in the study design. XD, PL, and LM implemented the field investigation and performed laboratory tests. JWa and WL participated in the study design and helped draft the manuscript. All authors contributed to the study and have read and approved the final manuscript.

## Funding

This study was funded by the National Natural Science Foundation of China (81973103), Nanjing Major Science and Technology Project (2021-11005), and Medical Research Project of Jiangsu Health Commission (ZDB2020013). The funding agencies had no role in the study design, data collection, analysis, decision to publish, or preparation of the manuscript.

## Conflict of interest

The authors declare that the research was conducted in the absence of any commercial or financial relationships that could be construed as a potential conflict of interest.

## Publisher's note

All claims expressed in this article are solely those of the authors and do not necessarily represent those of their affiliated organizations, or those of the publisher, the editors and the reviewers. Any product that may be evaluated in this article, or claim that may be made by its manufacturer, is not guaranteed or endorsed by the publisher.
